# A Comparison Between Written Assessment Methods: Multiple-choice and Short Answer Questions in End-of-clerkship Examinations for Final Year Medical Students

**DOI:** 10.7759/cureus.3773

**Published:** 2018-12-24

**Authors:** Fareeha Farooqui, Nadia Saeed, Sahira Aaraj, Muneeza A Sami, Muhammad Amir

**Affiliations:** 1 Surgery, Shifa Tameer-e-Millat University, Islamabad, PAK; 2 Internal Medicine, Shifa Tameer-e-Millat University, Islamabad, PAK; 3 Pediatrics, Shifa Tameer-e-Millat University, Islamabad, PAK; 4 Medical Education and Simulation, Shifa Tameer-e-Millat University, Islamabad, PAK

**Keywords:** mcq, saq, clerkship, written assessment

## Abstract

Introduction

An important aspect of a modern academic curriculum is assessment, which can be clinical and written. Written assessment includes both multiple-choice questions (MCQs) and short answer questions (SAQs). Debate continues as to which is more reliable. It is important to assess the correlation between the two different formats of written assessments, especially in the clinical subjects as they are different from the basic science subjects. Moreover, data are lacking in the correlation of the two formats of the written assessment in the clinical subjects. Therefore, we conducted this study to see the correlation between MCQs and SAQs in the end-of-clerkship examinations for final-year medical students.

Materials and methods

The end-of-clerkship written assessment results of the four disciplines of medicine, surgery, gynecology, and pediatrics were included. This was a retrospective correlational analytical study conducted at Shifa Tameer-e-Millat University, Islamabad, from 2013 to 2017. Data were analyzed using IBM SPSS Statistics for Windows, version 23.0 (IBM Corp., Armonk, NY); mean, standard deviation, Pearson coefficient, and p values were calculated both for MCQs and SAQs.

Results

A total of 481 students were involved in our study. The mean percentage scores of MCQs and SAQs in medicine were the most similar, and scores in obstetrics and gynecology had the most disparity. As compared to MCQs, the wider standard deviations were found in SAQs. Pearson correlations were 0.49, 0.47, 0.23, and 0.38 for medicine, surgery, gynecology, and pediatrics, respectively.

Conclusion

While we found mild to moderate significant correlation between MCQs and SAQs for final-year medical students, further investigations are required to explore the correlation and enhance the validity of our written assessments.

## Introduction

Medical education has dramatically changed over the past few decades [1]. The modern curriculum includes clerkships during the clinical years [2]. An important aspect of this modern curriculum is assessment [3-4]. Assessment is an essential learning tool to explore whether the objectives have been fulfilled [3]. For assessments, practical or written examinations can assess the student’s cognition, skill performance, and attitude [4]. For the undergraduate, clinical year clerkship cognition can be assessed in two different written formats: free response format (FRF) or selected response format (SRF) while skill performance and attitude are assessed in the clinical examination [4-5]. SRF includes multiple-choice questions (MCQs) and extended matching questions. FRF includes short answer questions (SAQs), short essay questions (SEQs), and long answer questions, and each of these assessment methods offers benefits, yet there is no consensus on which method is superior [5-6].

The MCQs have the advantage of being more objective and easily scored both manually and electronically [7]. Moreover, MCQs can also assess the problem-solving skills [8]. However, no assessment method is perfect, and MCQs have some disadvantages, too. For example, they have a cueing effect, so there are higher chances of student guessing, which ultimately lead to falsely higher scores than other methods [9]. Constructing good quality MCQs to warrant higher cognition is difficult and labor-intensive [10].

SAQ scoring is more time consuming and subjective than other MCQs, and hence, SAQs are prone to error and risks of bias. Sometimes, handwritten responses are ambiguous and illegible [7,11]. Moreover, unlike MCQs, grading metrics need to be structured in advance, and the answer key has to be developed for assessors to minimize the risk of bias. However, this format of examination reflects student’s interpretive skills and provides flexibility in their responses. Furthermore, it does not have the cueing effect of MCQs and can be used to assess problem-solving skills [8-9].

Currently, no single best assessment method with absolute reliability and validity exists. For a valid assessment, multiple methods should be employed. A correlation should exist between the assessment methods, targeting measures of the same trait [12]. Several studies have established a strong correlation between MCQs and SAQs [13], although MCQs with a high cognition level have a well-known superior validity and reliability [8,14].

We conducted this study to explore the correlation between the effectiveness and assessment properties of SAQs and MCQs in the written end-of-clerkship assessment at our medical college over the last five years. Because Shifa College of Medicine has a well-trained faculty and examination department, and since measures are taken to assure quality standards, there should be a significant correlation between the effectiveness of SAQs and MCQs. Our findings may help avoid duplication of written assessments and results in the future, which may help reduce unnecessary efforts for the faculty in writing both MCQs and SAQs to assess the same cognitive skills in a single subject.

## Materials and methods

This retrospective correlation analytical study was conducted at Shifa Medical College of Shifa Tameer-e-Millat University, Islamabad, Pakistan using end-of-clerkship scores from MCQs and SAQs of final year medical students from 2013 to 2017. The local Institutional Review Board's approval, reference number 1036-311-2018 was taken on May 2018. The data were obtained from the college examination department. To maintain privacy and confidentiality, names and roll numbers of students were not disclosed.

At Shifa College of Medicine, during final year clerkships, students were rotated in medicine, surgery, pediatrics and obstetrics and gynecology departments. Each clerkship consists of nine weeks of rotation in each discipline and the allied specialties. At the end of the ninth week, there was an end-of-clerkship examination including a written paper, MCQs, SAQs, and an objective structured clinical examination. Shifa College of Medicine has a well-trained faculty for writing MCQs and SAQs in each discipline. To improve the reliability and quality standards, all the MCQs and SAQs papers are constructed by well-trained senior faculty and vetted by the Department of Medical Education.

The surgery and medicine examination consisted of 100 MCQs, and each carried 100 marks. The pediatric and obstetrics and gynecology examinations contained 70 MCQs each, carrying 70 marks. MCQs had a single best answer among five options. There was no negative marking for a wrong response. A computer-based assessment of MCQs was performed according to the key using Remark Classic Optical Mark Reader software, version 2.5 (Gravic Inc., Malvern, PA).

Each paper included all three levels of MCQs according to Bloom’s taxonomy. The SAQ paper consisted of 10 questions with three marks for every question, making a total of 30 marks for each SAQ paper. In all disciplines, SAQs were graded manually according to a preformed rubric to minimize the risk of bias.

The reliability analysis of MCQs used data taken from college examination records and calculated by Cronbach’s alpha. Minimum, maximum, and standard deviations of obtained marks were calculated from percentage scores. Pearson’s correlation coefficient was used to assess the correlation between MCQs and SAQs percentage scores using IBM SPSS Statistics for Windows, version 23.0 (IBM, Corp., Armonk, NY).

## Results

Our analysis included SAQs and MCQs scores from 481 final-year MBBS students from 2013 to 2017. The minimum and maximum percentage scores of MCQs and SAQs in medicine, surgery, pediatrics, and obstetrics and gynecology are shown in Table 1. The least difference between the mean percentage scores of MCQs and SAQs was in medicine, and the largest difference was noted in obstetrics and gynecology. As compared to MCQs, wider standard deviations were found in SAQs. Except in medicine, students scored higher with MCQs than with SAQs. The correlation coefficient calculated through Pearson’s formula is shown in Table 2.

**Table 1:** Mean percentage scores for individual clerkships (n=481) SAQs: short-answer questions; MCQs: multiple-choice questions

Disciplines	Assessments	Minimum %	Maximum %	Mean	Standard Deviation
Medicine	SAQs	29	100	74.08	13.96
	MCQs	35	93	73.12	12.18
Obstetrics and Gynecology	SAQs	10	93	55.93	12.90
	MCQs	26	89	64.72	10.08
Pediatrics	SAQs	34	100	72.25	13.21
	MCQs	54	100	79.86	9.09
Surgery	SAQs	16	88	52.78	15.15
	MCQs	3	83	59.12	11.57

**Table 2:** Correlations between MCQs and SAQs (calculated from percentages scores, n=481) SAQs: short-answer questions; MCQs: multiple-choice questions

Disciplines	Pearson Correlation	P value
Medicine	0.495	0.00
Surgery	0.469	0.00
Obstetrics and Gynecology	0.228	0.00
Pediatrics	0.380	0.00

**Figure 1:**
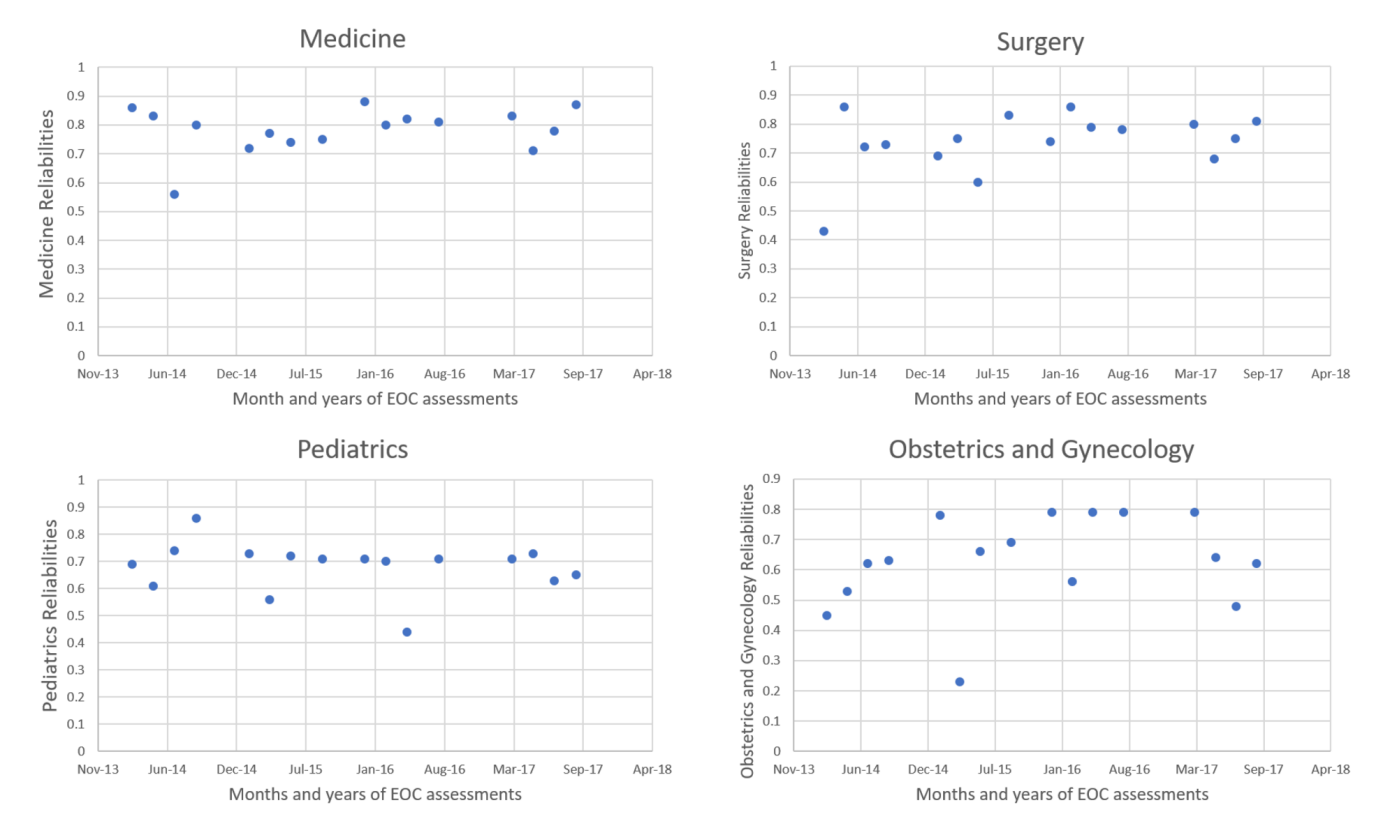
Reliabilities of multiple-choice questions in medicine, surgery, pediatrics, and obstetrics and gynecology (n= 481) EOC: end-of-clerkship

We found mild to moderate but significant correlations between MCQs and SAQs among all four disciplines. There is a moderately positive correlation between MCQ and SAQ in medicine, surgery, and pediatrics. There is a weak positive correlation in obstetrics and gynecology MCQs and SAQs (p<0.05 was considered significant). Figure 1 shows the reliabilities of MCQs in four disciplines over five years.

## Discussion

The aim of assessments is to determine the adequacy of knowledge with standardization [5,10]. Choosing the best method for assessment in terms of reliability and validity remains a matter of debate [15]. The attempt to change the current methods of assessment is hindered by a lack of data supporting one tool or another [16]. The efficacy of MCQs alone as an assessment tool has attracted considerable debate over the past few decades, and the same is true for SAQs [16]. Currently, many medical colleges are using both formats in undergraduate final exams. At Shifa College of Medicine, end-of-clerkship examinations comprise 15% of final professional examination scores in all four disciplines, and students take it seriously.

We found a statistically significant overall correlation between students’ performance on MCQ and SAQ in all the four major subjects. The students who performed well in SAQs were also likely to do well in MCQs. Mujeeb et al. and Pepple et al. noted a similar relationship in their students’ performance [10,17]. Many other studies compare MCQs to SEQs [13,17-18], but no study has compared MCQs and SAQs. SAQs are different from SEQs as the student has to write a single line answer to an SAQ rather than narrating it in few lines as for an SEQ [16].

The comparison of these two modalities has also been performed in various disciplines including anatomy, physiology, pharmacology, ophthalmology, and surgery [7,10,17-19]. Most of the studies reporting a correlation between the two formats focused on basic sciences. In pre-clinical years, Adeniyi et al. studied physiology examination results, and Pai et al. studied pharmacology results [18-19]. Moreover, studies on clinical subjects focus on only one subject at a time like Mahmood in 2015, who observed ophthalmology results for fourth-year students [7] and Dakum et al. [20] who studied results of surgery only. Our study is unique as it compares student performance in SAQs and MCQs in four clinical disciplines at one time and checks their correlation. Our most important finding was a mild to moderate significant correlation between MCQ and SAQs scores in all four disciplines.

Assessing students in basic or pre-clinical subjects requires a different approach than that used for clinical subjects, especially in problem-solving and clinical based scenarios. Creating such an exam is time-consuming and requires concentration and expertise [3,5]. Choosing one of two examination formats with strong positive correlation can reduce the work of faculty by 50%. It can also improve the quality of the examination.

We noted a moderately positive correlation between MCQ and SAQ in medicine, surgery, pediatrics, and a weakly positive correlation in obstetrics and gynecology. This difference in correlation cannot be attributed solely to faculty or paper content. The faculty in Shifa Tameer-e-Millat University is equally trained in writing MCQs and SAQs through regular workshops. Every paper is properly vetted by our Medical Education Department. We have a thorough post hoc analysis system, through which regular feedback is given to all the disciplines, and subsequent assessments are modified to improve the validity of the next assessment. The reliabilities of individual MCQs calculated through Cronbach’s alpha are usually between 0.6 to 0.8. However, our post-assessment analysis lacks correlation assessments. This is the first we have analyzed data from the last five years for this purpose, and correlation assessments between MCQs and SAQs should not be overlooked. Students’ sample remained similar throughout each year, so the variation in correlations and reliabilities calls to question the standards and quality assurance strategies and necessitates justification. It is important to investigate the reasons for students’ inconsistent performance in different clerkships. The detailed evaluation of MCQs and SAQs over the last five years would be beyond the scope for this study. However, our study necessitates a thorough evaluation of our clerkship objectives and removal of flaws in our facility’s written assessments.

A reason for this difference in correlation may be subject-dependent and gender-dependent [20-21]. Given that surgery and medicine are major subjects, students usually tend to concentrate more on these subjects. Male students might not focus on obstetrics and gynecology; male students do not interact with or examine obstetrics and gynecology patients which may lead to a failure to attempt reasoning and problem-solving questions in those fields. Because we do not have a gender-based analysis of correlation in each discipline, we cannot explore this further in the current study. However, this difference in correlation in obstetrics and gynecology calls for further research based on gender differences in student performance in all the subjects except medicine (where results were similar). This may be due to marking bias in SAQs papers. A single assessment modality used in clerkships will yield different results than what would be obtained by using both SAQs and MCQs. This contrasts with Adeniyi et al. who reported more failure in MCQs for first-year students in physiology likely due to negative marking as a fundamental part of their exam pattern [19]. Mujeeb et al. and Pepple et al. had results similar to ours [10,17].

The wider standard deviations in SAQs may be explained by bias or variable marking by different examiners whereas marking is more consistent in MCQs. The desired reliability of MCQ is 0.8 or higher [15]. The reliability charts demonstrate that medicine had better reliabilities of MCQs in the last five years [14].

## Conclusions

The mild to moderate correlation between MCQs and SAQs questions the utility of MCQs alone. Our results can be applied to the practical improvement of assessment practices. A prospective study comparing the results of individual students in each discipline to explore correlation further is warranted. Additional studies with more stringent methods are required to further explore the correlation between different assessment modalities in order to achieve quality in assessments.
